# Neglected tropical diseases and global burden of disease in China

**DOI:** 10.1186/s40249-017-0237-y

**Published:** 2017-02-03

**Authors:** Men-Bao Qian

**Affiliations:** 1 Key Laboratory on Biology of Parasite and Vector, Ministry of Health, Shanghai, 200025 China; 2 National Center for International Research on Tropical Diseases, Shanghai, 200025 China; 3 WHO Collaborating Center for Tropical Diseases, Shanghai, 200025 China; 4 National Institute of Parasitic Diseases, Chinese Center for Disease Control and Prevention, Shanghai, 200025 China

**Keywords:** China, Global burden of disease, Neglected tropical diseases

## Abstract

Recently, the mortality of 240 causes in China including a subnational analysis during 1990–2013 was published in *The Lancet*. This comprehensive analysis will undoubtedly impact policymaking regarding public health in China. However, it is unfavourable in some degree to neglected tropical diseases, which is the subject of this Letter to the Editor. Policymakers, especially those in less developed provinces of China, should fully consider the burden of neglected tropical diseases, which will benefit the control and final elimination of these diseases in the country.

**Electronic supplementary material:**

The online version of this article (doi:10.1186/s40249-017-0237-y) contains supplementary material, which is available to authorized users.

## Multilingual abstracts

Please see Additional file 1 for translations of the abstract into the six official working language of the United Nations.

## Background

**Fig. 1:**
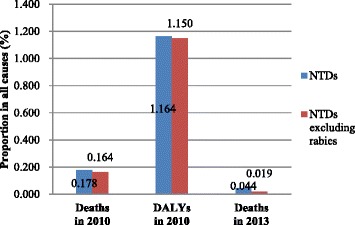
Proportion of NTDs in all causes in China by death and DALYs

Recently, Maigeng Zhou and colleagues released the mortality of 240 causes in China, which included a subnational analysis during 1990–2013, in *The Lancet* [1]. This comprehensive analysis will undoubtedly impact policymaking regarding public health in China. However, the analysis is unfavourable to neglected tropical diseases (NTDs). In Zhou’s article, the proportion of deaths caused by NTDs in all mortality causes in China in 2013 was only 0.044% (4 015/9 144 285) and only 0.019% (1 782/9 144 285) after excluding rabies (see Fig. 1) [1].

Although great achievements have been made in the control of NTDs during the past half-century in China, this recent report is likely to lead to the misconception about the unimportance of NTDs in contemporary China and subsequently to severe neglect in the control of these diseases.

### Why are these diseases neglected?

First, the inherent characteristics of NTDs determine their low mortality and high disability. According to Yang’s article on the Global Burden of Disease in China in 2010, the proportion of deaths caused by NTDs in all mortality causes was 0.178 and 0.164% after excluding rabies, while the corresponding disability-adjusted life years (DALYs) were 1.164 and 1.150%, respectively (see Fig. 1) [2]. The mortality indicator constitutes only a very small proportion of the life lost due to NTDs, as most NTDs do not cause significant death but rather chronic disability, which affects infected individuals as well as the socio-economics of the country in general.

Second, data on NTDs are usually underestimated due to inadequate reporting usually caused by the endemicity of NTDs in poor rural areas as well as insufficient research. For example, only 281 deaths caused by cystic echinococcosis were estimated in 2013 [1]. However, alveolar echinococcosis is usually co-endemic in areas with a presence of cystic echinococcosis. It was reported that about 25% echinococcosis is alveolar in China [3]. Further consideration of the higher severity of alveolar echinococcosis due to its progressive invasion, mortality cases due to alveolar echinococcosis could not be neglected. Another typical example is clonorchiasis. Clonorchiasis is currently the most important food-borne parasitic disease in China, but it was not classified as a definite carcinogen of cholangiocarcinoma until 2009 [4–6]. It is estimated that over 4 000 new cholangiocarcinoma cases are attributed to *Clonorchis sinensis* infection globally each year, about 85% of which occur in China [4]. Taking into account the high mortality of cholangiocarcinoma, the mortality due to *C. sinensis* infection is also likely to be significant.

Third, endemicity is also one typical characteristic of most NTDs. As demonstrated in Zhou’s article, the distribution of NTDs varies significantly at the provincial level and this imbalance in distribution also exists at the sub-provincial level. NTDs impact people in rural areas, especially in western China, who are affected by a combination of the presence of NTDs, poverty and inadequate medical services [7]. Thus, in a view of average level ignores the importance of NTDs in special areas.

Finally, it is important for interventions to be highly cost-effective at all times. Due to definite causes and effective interventions, control of NTDs always returns substantially, especially through integrated control packages.

### Suggestions

The control of NTDs is formally included in the Sustainable Development Goals [8]. Goal 3 aims to “ensure healthy lives and promote well-being for all at all ages”. Item 3 of Goal 3 stipulates ending the epidemics of NTDs by 2030, as well as of AIDS, tuberculosis and malaria. Thus, policymakers in China, especially in less developed provinces, should fully consider the burden of NTDs, including the associated high disability, low mortality, serious underreporting, and severe imbalance in their distribution and high cost-effectiveness in control. Furthermore, more research on NTDs is required, especially the establishment of a comprehensive database, which will ensure a more accurate evaluation of the burden of NTDs in China [9].

## Additional file

Additional file 1: Multilingual abstracts in the six officila working languages of the United Nations. (PDF 661 kb)

## Abbreviations

DALYs: Disability-adjusted life years
NTDs: Neglected tropical diseases
