# Supplementary material for: Neglected tropical diseases and global burden of disease in China
Source: Infect Dis Poverty. 2017 Feb 3;6:25. doi: 10.1186/s40249-017-0237-y (PMC5290607; doi:10.1186/s40249-017-0237-y)
Supplement: Additional file 1: — Multilingual abstracts in the six officila working languages of the United Nations. (PDF 661 kb) [file 40249_2017_237_MOESM1_ESM.pdf]

Translation of the abstract into the six official working languages of the United Nations

أمراض المناطق المدارية المهملة والعبء العالمي للمرض في الصين

مين-باو تشيان

ملخص

تم مؤخرا نشر 240 سببا للوفاة في الصين متضمنة تحليل دون الوطني أجري في الفترة من 1990 حتى 2013 في مجلة لانسيت. وهذا التحليل الشامل يؤثر بلا شك في السياسات المتعلقة بالصحة العامة في الصين. ومع ذلك، فهو غير متوافق إلى حد ما مع أمراض المناطق المدارية المهملة، والتي هي موضوع هذه الرسالة إلى محرر. صناع القرار، لا سيما في المحافظات الأقل نموا في الصين، ينبغي أن يهتموا بعبء الأمراض المدارية المهملة، والتي سوف تعود بالنفع على السيطرة والقضاء نهائيا على هذه الأمراض في البلاد.

Translated from English version into Arabic by Mahmoud Sami, through

TRANSLATORS
WITHOUT BORDERS

中国的被忽视热带病和全球疾病负担

钱门宝

摘要

近来，《柳叶刀》发表了中国全国及各省的 240 种死亡病因的分析。这一综合的分析毫无疑问将影响中国的公共卫生决策。然而，这一分析在某种程度上不利于被忽视热带病，这即是本文讨论的主题。中国的政策制定者，尤其是落后省份的，需要全面的看待被忽视热带病的疾病负担，这将有利于在中国控制和消除这类疾病。

Translated from English version into Chinese by Men-Bao Qian

Les maladies tropicales négligées et le fardeau global des maladies en Chine

Men-Bao Qian

Résumé

Récemment, un article sur la mortalité liée à 240 causes en Chine, comprenant une analyse infranationale de la période entre 1990 et 2013, a été publié dans *The Lancet*. Cette analyse exhaustive aura sans doute un impact sur l'élaboration de politiques touchant la santé publique en Chine. Toutefois, elle ne se penche pas, à un certain point, suffisamment sur les maladies tropicales négligées, soit le sujet de la lettre au rédacteur en chef. Les décideurs, surtout ceux des provinces moins développées de la Chine, devraient entièrement tenir compte du fardeau des maladies tropicales négligées afin d'aider à limiter la propagation de ces maladies au pays, et à les éliminer de manière définitiv1e.

Translated from English version into French by Edith Emilie Mercier, through

Забытые тропические болезни и глобальное бремя болезней в Китае

Men-Bao Qian

Автореферат

Недавно в журнале The Lancet были опубликованы коэффициенты смертности по 240 причинам смерти в Китае с субнациональным анализом за период 1990–2013 гг. Этот всеобъемлющий анализ, несомненно, повлияет на выработку политики в области общественного здравоохранения в Китае. Однако, в некоторой степени неблагоприятно скажется на забытых тропических болезнях, которые являются предметом настоящего письма в редакцию. Политическое руководство, особенно в менее развитых провинциях Китая, должно в полной мере оценить бремя забытых тропических болезней в целях обеспечения контроля и окончательной ликвидации этих болезней в стране.

Translated from English version into Russian by Anna Romanenko, through

Enfermedades tropicales desatendidas y su impacto negativo global en China

Autor: Men-Bao Qian

Resumen

Hace poco, *The Lancet* publicó un informe sobre las principales 240 causas de mortalidad en el mundo desde 1990 a 2013, que incluye un análisis a nivel de países, entre los cuales se encuentra China. Este exhaustivo análisis sin duda influirá en la legislación sobre salud pública en China; sin embargo, creemos que no considera suficientemente el impacto de las enfermedades tropicales desatendidas, tema de esta Carta al editor. Los legisladores en materia de salud pública, especialmente los de las provincias menos desarrolladas de China, deberían tomar en plena consideración las consecuencias negativas de las enfermedades tropicales desatendidas, cosa que favorecería el control y, en última instancia, la erradicación de dichas enfermedades en el país.

Translated from English version into Spanish by Marta Callava Linares, through
